# Optimization of combined microwave and hot air drying technology for purple cabbage by Response Surface Methodology (RSM)

**DOI:** 10.1002/fsn3.2444

**Published:** 2021-07-01

**Authors:** Jing Liu, Xiangli Li, Yanmin Yang, Haixiang Wei, Liping Xue, Min Zhao, Jinxiu Cai

**Affiliations:** ^1^ Jining Engineering and Technology Research Center for Special Agricultural Products High Value Processing Jining University Qufu China; ^2^ College of Food Science and Engineering Shandong Agricultural University Tai’an China; ^3^ Department of Food Science and Technology Shanghai Ocean University Shanghai China

**Keywords:** combined drying, drying quality, hot air drying, microwave drying, processing optimization, purple cabbage

## Abstract

Purple cabbage is one of the world's most widely consumed vegetables with high nutritional values containing the antioxidants and anti‐inflammatory activity of anthocyanins, vitamins, and minerals. But due to rapid postharvest quality decay, novel processing techniques including drying are required. In order to determine the conditions of combined microwave and hot air drying for purple cabbage, factors affecting the drying process including microwave density, hot air temperature, and the dry base water content at conversion point were investigated using the anthocyanin content, DPPH antioxidant capacity, chewiness, *△E*, rehydration ratio, and average drying rate as responses. The combined drying conditions were optimized considering three independent variables at three different levels by response surface methodology. The results showed that the processing parameters of purple cabbage with combined microwave and hot air drying technology were microwave density at 2.5 W/g, moisture content of conversion point at 4.0 g/g, and hot air temperature at 55°C. Under these conditions, the anthocyanin content, DPPH antioxidant capacity, chewiness, *△E*, rehydration ratio, average drying rate, and overall score of the dried purple cabbage were 175.87 mg/100 g, 87.59%, 4,521.468 g, 26.5, 4.3, 0.76 g/min, and 0.785, respectively. Therefore, combined microwave and hot air drying technology is an effective, suitable method for drying purple cabbage.

## Graphical abstract

In this paper, we study the effects of microwave density, hot air temperature, and moisture content at conversion point on the quality of purple cabbage. We also optimize the parameters of combined microwave and hot air drying by response surface methodology (RSM).

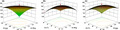

## 1 INTRODUCTION

Purple cabbage (*Brassica oleracea* L.), which has been widely used for therapeutic purposes for many years, is one of the most important vegetables grown around the world for consumption (Li et al., 2020; Mizgier et al., 2016; Wiczkowski et al., 2013). It has been reported that purple cabbage is highly suitable for obtaining natural dyes for food products due to the high content of anthocyanins (Ahmadiani et al., 2014; Podsedek, 2007). Anthocyanins have many beneficial effects, including the prevention of obesity and providing protection against cardiovascular diseases, cancer, and diabetes (Bakuradze et al., 2019). Besides, high concentrations of anthocyanins pigment do not present toxic, teratogenic, or mutagenic effects (Alvarez et al., 2019), so purple cabbage pigment is being used in beverages, chewing gum, candies, dry mixes, sauces, and yogurt (He, Zhang, Yue, et al., 2016; He et al., 2016). However, mechanical injury to vegetable after harvesting can generate a series of detrimental physiological and biochemical changes, such as enzymatic browning, nutrient content deterioration, and microbial contamination (Li et al., 2020).

Drying is one of the most important methods in food processing, and the major aim is to decrease water activity, which in turn minimizes the chemical and physical modifications during storage (Chen et al., 2017). Microwave drying has the advantage of uniform material heating and fast drying rate and is widely used in the drying of agricultural products (Ashtiani et al., 2018). However, microwave drying has been mainly used in the initial stage of preheating in sample drying because of high drying intensity and difficulties in power control (Ning et al., 2019). Hot air drying is very energy intensive, has low drying efficiency, and causes serious damage to the color, flavor, nutritional value, and rehydration capacity of the dried products (Ashtiani et al., 2018; Zhang et al., 2011). To overcome the limitations of only microwave drying or only hot air drying techniques, microwave‐hot air combination was used (Jebri et al., 2019; Wang et al., 2018).

A study (Chen et al., 2020) on combined microwave and hot air drying of celery stalk slices showed that it reduced drying time, minimized chromatic aberrations, and maximized the retention of the characteristic aroma of celery. Xu et al. (2018) investigated the effect of material temperature, temperature difference, and size of cube on the drying rate and quality of carrot cubes and determined the optimal drying condition of microwave combined with hot air drying. Also the obtained results by Yu et al. (2020) illustrated that the antityrosinase activity of saponins and polyphenols from asparagus could be enhanced by hot air coupled with microwave treatments. Das and Arora (2018) reported that the application of microwave combined hot air drying was more effective in shortening *Agaricus bisporus* mushroom drying time and improving product quality (low water activity, lighter in color, and higher rehydration ratio). Compared with numerous reports on fruits and vegetables, there are very few reports on the drying of purple cabbage. The objective of this paper was to examine the feasibility of using the combined microwave and hot air drying to dry purple cabbage. Through experiments, the effects of microwave density, switching point water content, and hot air temperature on anthocyanin content, DPPH antioxidant capacity, chewiness, *△E*, rehydration ratio, and average drying rate were examined. In addition, the parameters of combined microwave and hot air drying were optimized through response surface methodology (RSM) design to produce a high quality dried purple cabbage.

## 2 MATERIALS AND METHODS

### 2.1 Materials

Fresh purple cabbages were purchased from market and kept in cold storage at 4°C until further use. Slices of 1.5 cm × 3 cm squares were obtained by carefully cutting purple cabbages vertically with a vegetable slicer, and the slices were used for drying experiments. Drying experiments were accomplished in a microwave oven (NN‐CD997S; Panasonic Co., Ltd.) and in a blast drying box (101‐2AB; Taisite Instrument Co., Ltd.). The average initial water content of purple cabbages was recorded as 8.58 g/g in dry basis (db) utilizing the oven method at 105°C for 24 hr.

### 2.2 Drying experiments

The sliced samples, which were evenly distributed on the materials trays, were dried by different drying strategies until the final moisture content was less than 0.08 g/g (db). Microwave density, hot air temperature, and dry point moisture content at conversion point were chosen for studying the optimal technical conditions.

The cabbage slices were dried at various microwave densities (0.75, 1.75, 2.75, 3.75, 4.75 W/g, respectively) in NN‐CD997S microwave oven, with 1‐min interval every 1 min. Because the weighing took only a few seconds, it had little effect on the drying process (Wang et al., 2019; Yu et al., 2020). The cabbage slices were dried in 101‐2AB blast drying box at various temperatures (40, 50, 60, 70, or 80°C), with recirculation of air with 10%‐20% relative humidity and at approximately 1 m/s (Assis et al., 2018; Wang, Li, et al., 2019). The cabbage slices were also dried with a microwave density of 2.75 W/g reached the conversion point (30%, 40%, 50%, 60%, or 70%), and the microwave drying was replaced by hot air drying at 60°C. The optimization assay of response surface was designed such that the microwave density, moisture content at conversion point, and hot air temperature were considered as independent variables, while anthocyanin content, DPPH antioxidant capacity, chewiness, *△E*, rehydration ratio, and average drying rate were considered as response values.

### 2.3 Analysis of sample

#### 2.3.1 Moisture content

Moisture content was determined by the oven method. At regular time intervals during the drying processes, samples were taken out and dried at 105°C until constant weight (Zhang et al., 2011). The moisture content (db) was expressed as the ratio of the mass of water (g) to the mass of dry solid (g) (Wang et al., 2014). Average drying rate was expressed as the ratio of total mass reduction during drying (g) to the total drying time (min) (Horuz et al., 2018; Wang, Li, et al., 2019).

#### 2.3.2 Anthocyanin content

The dried samples were grounded by medicine grinder (FW177; Taisite Instrument Co., Ltd.) to a fine powder. The dried powder samples were weighed accurately of 250 mg. They were treated with 15 ml mixed solution of 95% ethanol and 1.5 mol/L HCl (85:15, pH1.0) and placed in a KH2200DV ultrasonic water bath at 100 W, 45°C (Hechuang Instrument Co., Ltd.) for 30 min. The extract was centrifuged for 5 min at 5,000 × g. The supernatants were collected, and the pellets were re‐extracted twice. The two extracts were combined and diluted to 100 ml (Hunaefi et al., 2013; Junka et al., 2017).

The pH differential technique of Giusti and Wrolstad (2001) with a slight modification was used for measuring anthocyanin content in this experiment. Potassium chloride buffer (0.02 mol/L KCl, pH 1.0) and sodium acetate (0.2 mol/L CH_3_CO_2_Na•3H_2_O, pH 4.5) were used for examining anthocyanin contents. One milliliter of extract from purple cabbage was added to the test tubes. Two tubes of one extract sample were followed by the two buffers. Nine milliliters of potassium chloride buffer and sodium acetate buffer was added. The test tubes were mixed and incubated for 60 min at room temperature (25°C). The anthocyanin was measured by spectrum scanning from 320 to 700 nm using a TU‐1900 visible spectrophotometer (General Instrument Co., Ltd). Anthocyanin content in the purple cabbage was calculated with Equation (1).

(1) $$\text{Anthocyanin content}\;\left(\text{mg}/100\;g\right)=\frac{[{\left(A_{\lambda \text{vis - max}}‐A_{\lambda 700}\right)}_{\text{pH}1.0}‐{\left(A_{\lambda \text{vis}‐\max}‐A_{\lambda 700}\right)}_{\text{pH}4.5}]\times M\times \text{DF}\times V\times 100}{\varepsilon \times L\times m_{f}}$$

where *A*_λvis‐max_ is the maximum absorbance of the mixed solution, *A*_λ700_ is the 700 nm absorbance of the mixed solution, M is cyanidin‐3‐glucoside which has molecular weight as 449.2 (g/mol), DF is the dilution factor, *V* is the total volume of anthocyanin extract (ml), ε is the molar absorptive as 26,900 (L/(mol cm)), *L* is the path length of the cuvette (cm), and *m_f_* is the weight of purple cabbage (g).

#### 2.3.3 DPPH antioxidant capacity

The dried powder samples (0.2 g) were added into 50% ethanol at the ratio of 1:50 (g:ml). After ultrasonic shock extraction at 45°C for 30 min, the extract was centrifuged for 15 min at 5,000 × g. The supernatants were collected, and the pellets were re‐extracted again. The two extracts were combined and diluted to 100 ml. Two milliliters ethanol extract was pipetted into a tube with 2 ml 0.1 mmol/L DPPH solution, and the mixture reacted in the dark at room temperature (25°C) for 30 min. The absorbance (*A*_i_) at 517 nm was determined using absolute ethanol as reference. The absorbance (*A*_0_) of the mixture of 2 ml absolute ethanol and 2 ml 0.1 mmol/L DPPH, and the absorbance (*A*_j_) of the mixture of 2 ml absolute ethanol and 2 ml ethanol extract were also detected, respectively. DPPH antioxidant capacity was calculated with Equation (2) (Szewczyk et al., 2020).

(2) $$\text{DPPH antioxidant capacity}\%=\left(1‐\frac{A_{i}‐A_{j}}{A_{0}}\right)\times 100$$

#### 2.3.4 Texture analysis

Chewiness mainly reflects the continuous resistance of the samples during chewing, which is positively correlated with hardness. According to the improved method of Rajkumar et al. (2016), after purple cabbage was rehydrated, drained, and sliced into 1.5 cm × 1.5 cm squares, the chewiness of 4 tablets was determined by TA‐XT.EXDRESS Enhanced Texture Analyzer (Stable Micro Systems Ltd.). Eight samples were used in each treatment. The cylinder penetrometer probe (P5) was passed through the samples with the test parameters set as: 1 mm/s of prespeed, 5 mm/s of test speed and postspeed, 70% compression ratio, and 5 g trigger.

#### 2.3.5 Total color change

The CIE L^*^a^*^b^*^ color coordinates were measured using a WSD‐3C automatic whiteness meter (Kangguang Instrument Co., Ltd.). The instrument was calibrated using standard white tile before the measurements. The surface color of the samples was measured in terms of L (degree of darkness), a (degree of redness and greenness), and b (degree of yellowness and blueness) (Omari et al., 2018). Samples were placed on the measure head of whiteness meter, and measurements of color were performed for all prepared samples. Finally, the total color change between blank white (L_0_, a_0_ and b_0_) and dried cabbage samples (L^*^, a^*^ and b^*^) was determined according to Equation (3) (Wang et al., 2020).

(3) $$\Delta E=\sqrt{{\left(L_{0}‐L^{*}\right)}^{2}+{\left(\alpha_{0}‐\alpha^{*}\right)}^{2}+{\left(b_{0}‐b^{*}\right)}^{2}}$$

#### 2.3.6 Rehydration ratio

The dried samples were immersed in 60°C distilled water for 30 min and drained on the filter paper for 30 min to remove free water on the surface. The rehydration ratio was expressed as the ratio of the mass rehydrated sample (g) to the mass of the dried sample (g) (Ashtiani et al., 2018).

#### 2.3.7 Overall score

According to the method of Hu et al. (2006), the overall score of the samples was calculated based on the sum of the weight coefficient of each index and the membership function value (*l*) of tested values. Based on the emphasis of nutritive value, rehydration performance, and drying speed of the dry samples, the weight coefficient was given to calculate the overall score, including anthocyanin content (0.25), DPPH antioxidant capacity (0.2), rehydration ratio (0.2), *△E* (0.15), chewiness (0.1), and drying rate (0.1). The lower the chewiness, the lower the hardness of the dried samples, and the better of the taste (Zhou et al., 2020). The chewiness function and the total color change were calculated by $l=\frac{l_{\text{max}}‐l_{i}}{l_{\text{max}}‐l_{\text{min}}}$. But the higher the other indicators, the better the quality. The membership function was calculated by $l=\frac{l_{i}‐l_{\text{min}}}{l_{\text{max}}‐l_{\text{min}}}$.

The *l*_i_ refers to the index tested value, *l*_max_ refers to the maximum value of the index tested value, and *l*_min_ refers to the minimum value of the index tested value, and the higher the *l* value, the better the quality of dried products.

### 2.4 Data processing

Each experiment was repeated three replicates, and graphs were drawn with Microsoft Excel 2013. The mean values for all parameters were examined for significance by analysis of variance using SPSS Statistics 22.0 software. *p* < .05 meant a significant difference, and *p* < .01 meant a highly significant difference. Design‐Expert 8.0.7.1 software (Stat‐Ease) was used to analyze the results of the response surface assay.

## 3 RESULTS AND DISCUSSION

### 3.1 Single factor experiment of combined microwave and hot air drying

**FIGURE 1:**
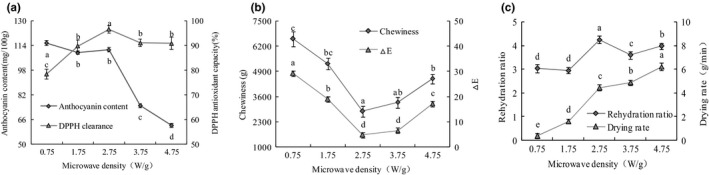
Effect of microwave density on the quality of purple cabbage. (a) Anthocyanin content and DPPH antioxidant capacity; (b) chewiness and *△E*; (c) rehydration ratio and drying rate

The influences of microwave density on anthocyanin content, DPPH antioxidant capacity, chewiness, *△E*, rehydration ratio, and drying rate were shown in Figure 1. As microwave density increased from 0.75 to 4.75 W/g, drying rate increased and reached its maximum value of 6.23 g/min at 4.75 W/g while the anthocyanin content decreased and had a minimum value about 62.31 mg/100 g at 4.75 W/g. As the microwave density increased, the DPPH antioxidant capacity and rehydration ratio first increased and then decreased, reaching the highest value of 96.5% and 4.26 at 2.75 W/g, respectively, whereas the chewiness and *△E* initially decreased rapidly and then increased slowly as microwave density increased. When the microwave density was 2.75 W/g, the chewiness and *△E* reached its minimum value of 2,838.2 g and 4.7, respectively. All these results showed that the purple cabbage dried at 1.75 ~ 3.75 W/g had a better drying quality except *△E*. Since a lower microwave density needed a longer drying period, the dried products showed poor DPPH antioxidant capacity (Wang, Li, et al., 2019). An increase in the microwave density gradually resulted in a porous dried product with severe browning, improved rehydration and good DPPH antioxidant capacity (Isik, 2014). However, when the microwave density was too high, the cell wall tissue of purple cabbage was destroyed. Moreover, the rapid dehydration caused a burning phenomenon, improving the color *△E* and reducing sensory quality (Maray et al., 2017). These were consistent with a previous report for macadamia drying and unripe grapes drying (Pakonkiad et al., 2018; Razgeh et al., 2020).

**FIGURE 2:**
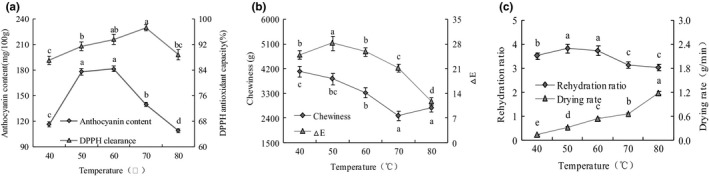
Effect of hot air temperature on the quality of purple cabbage. (a) Anthocyanin content and DPPH antioxidant capacity; (b) chewiness and *△E*; (c) rehydration ratio and drying rate

The influences of hot air drying temperature on anthocyanin content, DPPH antioxidant capacity, chewiness, *△E*, rehydration ratio, and drying rate were shown in Figure 2. As hot air temperature increased from 40 to 80°C, drying rate increased and reached its maximum value of 1.18 g/min at 80°C. As the hot air temperature increased, the anthocyanin content, DPPH antioxidant capacity, *△E*, and rehydration ratio initially increased and then decreased, reaching the highest value of 181.15 mg/100 g at 60°C, 97.2% at 70°C, 28.3 at 50°C, and 3.84 at 50°C, respectively, whereas the chewiness first decreased and then increased as the hot air temperature increased. When the hot air temperature was 70°C, the chewiness reached its lowest value of 2,495.28 g. These results showed that the purple cabbage dried at 50 ~ 70°C had a better drying quality than the other treatments. This was due to the moisture evaporation on the materials surface was slow at low hot air temperature, and the enzymatic browning reaction catalyzed by polyphenol oxidase (Assis et al., 2018; Wang, Liu, et al., 2019). When the hot air temperature increased, the antioxidant substances in the cabbages, including anthocyanin, polyphenols, and flavonoids, caused severe heat damages and intensified the Maillard reaction (Xia, 2013). The similar behaviors were also reported in apple slices, *Asparagus officinalis*, white button mushroom slices, and potato chips (Baltacioglu, 2017; Doymaz, 2014; Horuz et al., 2018; Luo et al., 2019).

**FIGURE 3:**
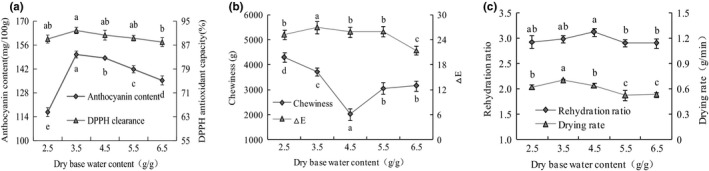
Effect of dry base water content on the quality of purple cabbage. (a) Anthocyanin content and DPPH antioxidant capacity; (b) chewiness and *△E*; (c) rehydration ratio and drying rate

The influences of dry point moisture content at conversion point on anthocyanin content, DPPH antioxidant capacity, chewiness, *△E*, rehydration ratio, and drying rate were shown in Figure 3. As dry base water content increased from 2.5 to 6.5 g/g, anthocyanin content, DPPH antioxidant capacity, *△E*, rehydration ratio, and drying rate initially increased and then decreased, while the results of chewiness was opposite. The anthocyanin content, DPPH antioxidant capacity, *△E*, and drying rate at 3.5 g/g dry base water content had the maximum value of 150.47 mg/100 g, 91.86%, 26.9, and 0.70 g/min, respectively. When the dry point moisture content at conversion point was 4.5 g/g, the chewiness reached the lowest value of 2,005.19 g, but the rehydration ratio showed the highest value of 3.12. All these results showed that the purple cabbage dried under 3.5 ~ 4.5 g/g moisture content at conversion point could have a better drying quality and most of quality indexes were better than the others. This may be because microwave drying was beneficial to maintain the shape of cabbages and gave the dried product a reducing anthocyanin content, while hot air drying made the products shrink severely (Maray et al., 2017; Yadav et al., 2017). Similar results were also obtained by Wang, Li, et al. (2019) and Razgeh et al. (2020), indicating that hot air combined with microwave should be considered due to its obviously enhanced physical properties and chemical components.

### 3.2 Optimization of combined microwave and hot air drying

#### 3.2.1 The experimental design and results of response surface

The most important variables in combined microwave and hot air drying process, that is, microwave density (*X*_1_, 1.75–3.75 W/g), dry point moisture content at conversion point (*X*_2_, 3.5–4.5 g/g), and hot air temperature (*X*_3_, 50–70°C), were optimized using response surface methodology (RSM). Results of investigated responses, including anthocyanin content (*Y*_1_), DPPH antioxidant capacity (*Y*_2_), chewiness (*Y*_3_), *△E* (*Y*_4_), rehydration ratio (*Y*_5_), and drying rate (*Y*_6_), obtained in designed experiments using Box–Behnken design (BBD), Design‐Expert 8.0.7.1 software, were presented in Table 1.

**TABLE 1:** Results of combined microwave and hot air drying of purple cabbage in response surface methodology

Number	*X*_1_ microwave density (W/g)	*X*_2_ Dry base water content (g/g)	*X*_3_ Temperature (°C)	*Y*_1_ Anthocyanin content (mg/100 g)	*Y*_2_ DPPH antioxidant capacity (%)	*Y*_3_ Chewiness (g)	*Y*_4_ *△E*	*Y*_5_ Rehydration ratio	*Y*_6_ Drying rate (g/min)
1	1 (3.75)	1 (4.5)	0 (60)	162.84	83.81	5,863.56	25.9	3.47	0.44
2	0 (2.75)	−1 (3.5)	1 (70)	124.76	87.30	2,826.50	23.6	3.67	0.74
3	1	0(4.0)	−1 (50)	167.84	79.80	4,172.92	26.9	3.69	0.50
4	−1 (1.75)	0	1	127.50	86.56	5,401.50	25.6	3.60	0.88
5	1	0	1	158.04	83.87	3,802.38	24.6	3.59	0.69
6	0	0	0	159.24	86.98	3,773.11	26.7	4.08	0.77
7	0	0	0	159.59	85.76	3,873.11	27.3	4.18	0.67
8	0	1	1	116.29	91.80	3,850.39	24.0	2.94	0.63
9	−1	−1	0	153.48	83.67	6,084.27	24.9	4.13	0.68
10	0	−1	−1	190.94	84.10	3,991.95	27.5	3.47	0.60
11	0	0	0	175.20	88.46	3,688.46	26.8	4.15	0.68
12	0	0	0	162.07	88.26	3,764.94	28.1	4.23	0.69
13	−1	1	0	172.34	85.68	5,181.17	26.1	4.09	0.79
14	−1	0	−1	179.05	84.03	5,615.70	26.7	4.50	0.58
15	0	0	0	161.29	88.59	3,026.43	27.6	4.26	0.70
16	0	1	−1	183.95	82.31	3,489.86	27.0	3.93	0.49
17	1	−1	0	185.58	86.26	4,474.92	28.5	3.23	0.63

#### 3.2.2 Establishment and variance analysis of regression equation

According to the Box–Behnken principle, Design‐Expert 8.0.7.1 software was used to do quadratic polynomial regression analysis toward the results in Table 1 to obtain the following 6 ternary quadratic regression equations:

$$Y_{1}=‐53.05+0.50X_{1}+41.45X_{2}+6.95X_{3}‐20.80X_{1}X_{2}+1.04X_{1}X_{3}‐0.074X_{2}X_{3}+4.60X_{1}^{2}+1.92X_{2}^{2}‐0.10X_{3}^{2}$$

$$Y_{2}=+65.69‐21.15X_{1}‐13.09X_{2}+0.39X_{3}‐2.23X_{1}X_{2}+0.039X_{1}X_{3}+0.31X_{2}X_{3}‐2.78X_{1}^{2}+0.11X_{2}^{2}‐0.013X_{3}^{2}$$

$$Y_{3}=51,453.06‐13,052.20X_{1}‐16,557.38X_{2}+131.10X_{3}+1,145.87X_{1}X_{2}‐3.91X_{1}X_{3}+76.30X_{2}X_{3}+1,492.20X_{1}^{2}+1,135.02X_{2}^{2}‐3.69X_{3}^{2}$$

$$Y_{4}=64.55+11.17X_{1}+24.15X_{2}+1.08X_{3}‐1.90X_{1}X_{2}‐0.03X_{1}X_{3}+0.045X_{2}X_{3}‐0.26X_{1}^{2}‐2.75X_{2}^{2}‐0.011X_{3}^{2}$$

$$Y_{5}=‐38.93‐1.76X_{1}+15.85X_{2}+0.50X_{3}+0.14X_{1}X_{2}+0.02X_{1}X_{3}‐0.06X_{2}X_{3}‐0.054X_{1}^{2}‐1.58X_{2}^{2}‐2.813E‐003X_{3}^{2}$$

$$Y_{6}=‐6.26+0.73X_{1}+2.17X_{2}+0.053X_{3}‐0.15X_{1}X_{2}‐2.75E‐003X_{1}X_{3}‐9.75E‐003X_{1}^{2}‐0.23X_{2}^{2}‐2.975E‐004X_{3}$$

As shown in Table 2, the correlation coefficients *R^2^* of models were close to 1 (range 0.8333–0.9594), indicating that the model correlations were good. The coefficients of variation (CV) were between 1.93% and 8.95%, and since the smaller the coefficient of variation, the higher the confidence, this indicated that the obtained model had high confidence in anthocyanin content, chewiness, drying rate, and extremely high confidence in other response values. These models obtained in this experiment could be used to predict each related index of purple cabbage during combined microwave and hot air drying.

**TABLE 2:** Analysis of variance of regression equations for each response value

Source	*Y*_1_	*Y*_2_	*Y*_3_	*Y*_4_	*Y*_5_	*Y*_6_
Model	0.0149*	0.0314*	0.0023**	0.0435*	0.0004***	0.0044**
*X*_1_	0.9938	0.0590	0.0006***	0.0583	0.0461*	0.0318*
*X*_2_	0.8309	0.6543	0.0383*	0.1451	0.0002***	0.0330*
*X*_3_	0.4121	0.7529	0.6507	0.1296	0.0009***	0.1738
*X*_1_ *X*_2_	0.1014	0.2190	0.0203*	0.0650	0.3093	0.0172*
*X*_1_ *X*_3_	0.1004	0.6552	0.8444	0.5122	0.0166*	0.2926
*X*_2_ *X*_3_	0.9484	0.0986	0.0871	0.6206	0.0023**	1.0000
*X*_1_^2^	0.4204	0.0106*	<0.0001***	0.5550	0.4164	0.6913
*X*_2_^2^	0.9314	0.9725	0.1729	0.1486	0.0004***	0.0454*
*X*_3_^2^	0.1060	0.1611	0.0890	0.0371*	0.0027**	0.2471
Lack of Fit	0.0753	0.1606	0.3185	0.1097	0.0530	0.2380
*R* ^2^	0.8823	0.8504	0.9336	0.8333	0.9594	0.9193
CV%	6.85	1.93	8.95	3.30	3.33	7.36

Note

***, **, and * denote 0.1%, 1%, and 5% levels of significance, respectively.

The factors affecting DPPH antioxidant capacity, chewiness, and drying rate were microwave density >dry point moisture content at conversion point >hot air temperature, among which microwave density had an extremely significant effect on the chewiness (*p* < .001) but had a significant effect on drying rate (*p* < .05), while the dry point moisture content at conversion point had a significant effect on chewiness and drying rate (*p* < .05). The factors affecting anthocyanin content were hot air temperature >dry point moisture content at conversion point >microwave density, but none reached statistical significance (*p* > .05). The factors affecting *△E* were microwave density >hot air temperature >dry point moisture content at conversion point, but none reached statistical significance (*p* > .05). The factors affecting rehydration ratio were dry point moisture content at conversion point >hot air temperature >microwave density, among which dry point moisture content at conversion point and hot air temperature had an extremely significant effect (*p* < .001), while microwave density had a significant effect (*p* < .05).

In order to determine the conditions of purple cabbage by combined microwave and hot air drying, a weighting processing of anthocyanin content, DPPH antioxidant capacity, chewiness, *△E*, rehydration ratio, and drying rate was adopted to calculate the overall score according to the method of Hu et al. (2006). The overall score results were shown in Table 3. The overall score model was significant (*p* = .0016, *p* < .01), and there was no lack of fit (*p* = .4001, *p* > .05), which indicated that the model was very significant and the model error was small with practical application significance. The factors affecting overall score were hot air temperature >microwave density >dry point moisture content at conversion point, and hot air temperature had a significant effect on the overall score (*p* < .01). The ternary quadratic regression equation was *Y* = 0.72 – 0.034*X*_1_ – 0.023*X*_2_ – 0.088*X*_3_ – 0.11*X*_1_ *X*_2_ + 0.057*X*_1_ *X*_3_ – 0.025*X*_2_ *X*_3_ – 0.095*X*_1_^2^ – 0.10*X*_2_^2^ – 0.13*X*_3_^2^, (*R^2^* = .9409), showing a good fit between the test value and the predicted value, and could be used to predict the overall score of purple cabbage during a combined microwave and hot air drying.

**TABLE 3:** Variance analysis of overall score regression model

Source	Sum of squares	*df*	Mean square	*F* value	*p*‐Value	Significance
Model	0.30	9	0.033	12.38	.0016	**
*X*_1_	8.999E‐003	1	8.999E‐003	3.34	.1103	
*X*_2_	4.166E‐003	1	4.166E‐003	1.55	.2537	
*X*_3_	0.061	1	0.061	22.81	.0020	**
*X*_1_ *X*_2_	0.045	1	0.045	16.85	.0045	**
*X*_1_ *X*_3_	0.013	1	0.013	4.90	.0425	*
*X*_2_ *X*_3_	2.486E‐003	1	2.486E‐003	0.92	.3687	
*X*_1_^2^	0.038	1	0.038	14.16	.0070	**
*X*_2_^2^	0.043	1	0.043	15.86	.0053	**
*X*_3_^2^	0.067	1	0.067	24.72	.0016	**
Residual	0.019	7	2.694E‐003			
Lack of Fit	9.162E‐003	3	3.054E‐003	1.26	.4001	
Pure Error	9.694E‐003	4	2.424E‐003			
			*R*^2 ^= .9409			

Note

** and * denote 1% and 5% levels of significance, respectively.

#### 3.2.3 Interaction analysis

**FIGURE 4:**
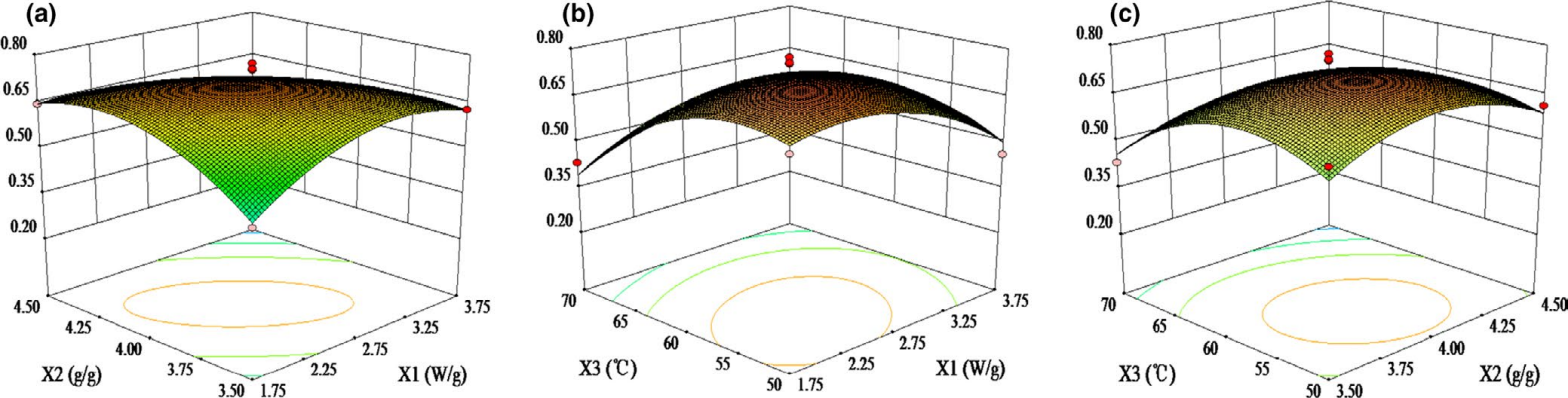
The effect of 3 factors on the overall score of purple cabbage by combined microwave and hot air drying. (a) Microwave density (*X*_1_) and dry point moisture content at conversion point (*X*_2_); (b) microwave density (*X*_1_) and hot air temperature (*X*_3_); (c) dry point moisture content at conversion point (*X*_2_) and hot air temperature (*X*_3_)

As shown in Figure 4, the opening of the response surface graph for the overall score was facing down, and when one variable was fixed, the overall score showed an increasing trend first followed by a decrease with the increase of another variable. The interaction of microwave density and dry point moisture content at conversion point showed a very significant difference (*p* < .01), and the interaction of microwave density and hot air temperature showed a significant difference (*p* < .05). When the hot air temperature was 60°C, the overall score had a response peak with a microwave density of 2.60 W/g and with a dry point moisture content at conversion point of 3.98 g/g. As the hot air temperature increased, the overall score increased to a maximum response value and then dropped rapidly. However, the response surface graph of the dry point moisture content at conversion point was relatively flat, which was consistent with the results of variation analysis indicating that the effect of hot air temperature was more important than that of the dry point moisture content at conversion point.

#### 3.2.4 Optimization and verification of combined microwave and hot air drying

According to the response surface analysis, the optimized processing parameters of purple cabbage by combined microwave and hot air drying were microwave density at 2.35 W/g, moisture content of conversion point at 4.08 g/g, and hot air temperature at 55.45°C. Based on the practical operability, we selected microwave density at 2.5 W/g, moisture content of conversion point at 4.0 g/g, and hot air temperature at 55°C as processing parameters to carry out three verification experiments of technical amplification. The results showed that the anthocyanin content, DPPH antioxidant capacity, chewiness, *△E*, rehydration ratio, and average drying rate of the dried purple cabbage were 175.87 mg/100 g, 87.59%, 4,521.468 g, 26.5, 4.3, and 0.76 g/min, respectively. The overall score was 0.785 and showed no significant difference from the predicted value of 0.797 (*p* > .05). In summary, the processing parameters for combined microwave and hot air drying of purple cabbage as identified in this work were accurate, reliable, and high practical value.

## 4 CONCLUSION

The factors affecting the overall score of dried purple cabbage products were hot air temperature, microwave density, and dry point moisture content at conversion point, and hot air temperature had a significant effect on the overall score (*p* < .01). The optimized parameters of combined microwave and hot air drying for purple cabbage were microwave density at 2.5 W/g, moisture content of conversion point at 4.0 g/g, and hot air temperature at 55°C. Under these conditions, the anthocyanin content, DPPH antioxidant capacity, chewiness, *△E*, rehydration ratio, drying rate, and overall score of the dried purple cabbage were 175.87 mg/100 g, 87.59%, 4,521.468 g, 26.5, 4.3, 0.76 g/min, and 0.785, respectively.

## CONFLICTS OF INTEREST

The authors have declared no conflicts of interest for this article.

## AUTHOR CONTRIBUTIONS

**Jing Liu:** Funding acquisition (lead); Investigation (lead); Resources (lead); Writing‐original draft (lead); Writing‐review & editing (lead). **Xiangli Li:** Funding acquisition (supporting); Methodology (supporting); Project administration (lead); Validation (lead); Writing‐original draft (equal); Writing‐review & editing (equal). **Yanmin Yang:** Formal analysis (equal); Methodology (equal); Project administration (supporting); Resources (supporting); Software (lead); Writing‐original draft (supporting); Writing‐review & editing (supporting). **Haixiang Wei:** Formal analysis (supporting); Investigation (equal); Methodology (equal); Project administration (supporting); Software (supporting); Visualization (supporting); Writing‐original draft (supporting); Writing‐review & editing (supporting). **Liping Xue:** Formal analysis (supporting); Methodology (equal); Resources (equal); Writing‐original draft (supporting); Writing‐review & editing (supporting). **Min Zhao:** Formal analysis (equal); Methodology (supporting); Resources (supporting); Writing‐original draft (supporting); Writing‐review & editing (supporting). **Jinxiu Cai:** Investigation (supporting); Methodology (equal); Project administration (supporting); Resources (equal); Writing‐original draft (supporting); Writing‐review & editing (supporting).

## ETHICAL APPROVAL

This study does not involve any human or animal testing.

## Data Availability

The data that support the findings of this study are available from the corresponding author upon reasonable request.

## References

[fsn32444-bib-0001] Ahmadiani, R., Robbins, J., Collins, T. M., & Giusti, M. M. (2014). Anthocyanins contents, profiles, and color characteristics of red cabbage extracts from different cultivars and maturity stages. Journal of Agricultural and Food Chemistry, 62, 7524–7531. 10.1021/jf501991q 24991694

[fsn32444-bib-0002] Alvarez, C. E., Contreras, J. L., Rodriguez, D. E., Rondon, D. J., Munoz, W. B., & Mezquita, P. C. (2019). Application of microencapsulated anthocyanin extracted from purple cabbage in fermented milk drinks. Acta Agronómica, 68(2), 134–141. 10.15446/acag.v68n2.79078

[fsn32444-bib-0003] Ashtiani, S. H. M., Sturm, B., & Nasirahmadi, A. (2018). Effects of hot‐air and hybrid hot air‐microwave drying on drying kinetics and textural quality of nectarine slices. Heat and Mass Transfer, 54(4), 915–927. 10.1007/s00231-017-2187-0

[fsn32444-bib-0004] Assis, F. R., Morais, R. M. S. C., & Morais, A. M. M. B. (2018). Rehydration of smoticlly pre‐treated apple cubes dried by hot air, microwave, and freeze‐drying. Acta Alimentaria, 47(3), 315–323. 10.1556/066.2018.47.3.7

[fsn32444-bib-0005] Bakuradze, T., Tausend, A., Galan, J., Maria Groh, I. A., Berry, D., Tur, J. A., Marko, D., & Richling, E. (2019). Antioxidative activity and health benefits of anthocyanin‐rich fruit juice in healthy volunteers. Free Radical Research, 53(1), 1045–1055. 10.1080/10715762.2019.1618851 31088176

[fsn32444-bib-0006] Baltacioglu, C. (2017). Optimization of drying and osmotic dehydration of *Asparagus officinalis* in microwave and conventional hot air oven using response surface methodology. Carpathian Journal of Food Science and Technology, 9(3), 5–16.

[fsn32444-bib-0007] Chen, G., Wu, F., Pei, F., Cheng, S., Muinde, B., Hu, Q., & Zhao, L. (2017). Volatile components of white Hypsizygus marmoreus detected by electronic nose and HS‐SPME‐GC‐MS: Influence of four drying methods. International Journal of Food Properties, 20, 2901–2910. 10.1080/10942912.2016.1258575

[fsn32444-bib-0008] Chen, Y., Song, C., Li, Z., Chen, H., & Jin, G. (2020). Effects of hot air and combined microwave and hot air drying on the quality attributes of celery stalk slices. Journal of Food Processing and Preservation, 44(1), e14310. 10.1111/jfpp.14310

[fsn32444-bib-0009] Das, I., & Arora, A. (2018). Alternate microwave and convective hot air application for rapid mushroom drying. Journal of Food Engineering, 223(4), 208–219. 10.1016/j.jfoodeng.2017.10.018

[fsn32444-bib-0010] Doymaz, I. (2014). Drying kinetics and rehydration characteristics of convective hot‐air dried white button mushroom slices. Journal of Chemistry, 2014, 1–8. 10.1155/2014/453175

[fsn32444-bib-0011] Giusti, M. M., & Wrolstad, R. E. (2001). Characterization and measurement of anthocyanins by UV‐visible spectroscopy. Current Protocols in Food Analytical Chemistry, 8, 1–13. 10.1002/0471142913.faf0102s00

[fsn32444-bib-0012] He, B., Zhang, L., Yue, X., Liang, J., Jiang, J., Gao, X., & Yue, P. (2016). Optimization of ultrasound assisted extraction of phenolic compounds and anthocyanins from blueberry (*Vaccinium ashei*) wine pomace. Food Chemistry, 204(1), 70–76. 10.1016/j.foodchem.2016.02.094 26988477

[fsn32444-bib-0013] He, Q., Zhang, Z., & Zhang, L. (2016). Anthocyanin accumulation, antioxidant ability and stability, and a transcriptional analysis of anthocyanin biosynthesis in purple heading Chinese cabbage (*Brassica rapa* L. ssp. *pekinensis*). Journal of Agricultural and Food Chemistry, 64(1), 132–145, 10.1021/acs.jafc.5b04674 26709726

[fsn32444-bib-0014] Horuz, E., Bozkurt, H., Karatas, H., & Maskan, M. (2018). Simultaneous application of microwave energy and hot air to whole drying process of apple slices: Drying kinetics, modeling, temperature profile and energy aspect. Heat and Mass Transfer, 54, 425–436. 10.1007/s00231-017-2152-y

[fsn32444-bib-0015] Hu, Q. G., Zhang, M., Mujumdar, A. S., Xiao, G. N., & Jin, C. S. (2006). Drying of edamames by hot air and vacuum microwave combination. Journal of Food Engineering, 77, 977–982. 10.1016/j.jfoodeng.2005.08.025

[fsn32444-bib-0016] Hunaefi, D., Gruda, N., Riedel, H., Akumo, D. N., Saw, N. M., & Smetanska, I. (2013). Improvement of antioxidant activities in red cabbage sprouts by lactic acid bacterial fermentation. Food Biotechnology, 27(4), 279–302. 10.1080/08905436.2013.836709

[fsn32444-bib-0017] Isik, N. I. E. (2014). Effect of different drying methods on drying characteristics, colour and microstructure properties of mushroom. Journal of Food and Nutrition Research, 53, 105–116.

[fsn32444-bib-0018] Jebri, M., Desmorieux, H., Maaloul, A., Saadaoui, E., & Romdhane, M. (2019). Drying of *Salvia officinalis* L. by hot air and microwaves: Dynamic desorption isotherms, drying kinetics and biochemical quality. Heat and Mass Transfer, 55, 1143–1153. 10.1007/s00231-018-2498-9

[fsn32444-bib-0019] Junka, N., Rattanamechaiskul, C., Wongs, A. C., & Kanlayanarat, S. (2017). Comparative study of organic solvents and extraction conditions on colour and antioxidant capacity in red cabbage. International Food Research Journal, 24(2), 518–524.

[fsn32444-bib-0020] Li, H., Li, X., Wang, R., Xing, Y., Xu, Q., Shui, Y., Guo, X., Li, W., Yang, H., Bi, X., & Che, Z. (2020). Quality of fresh‐cut purple cabbage stored at modified atmosphere packaging and cold‐chain transportation. International Journal of Food Properties, 23(1), 138–153. 10.1080/10942912.2020.1716795

[fsn32444-bib-0021] Luo, G., Song, C., Pu, H., Li, Z., Xu, W., Raghavan, G. S. V., Chen, H., & Jin, G. (2019). Optimization of the microwave drying process for potato chips based on the measurement of dielectric properties. Drying Technology, 37(11), 1329–1339. 10.1080/07373937.2018.1500482

[fsn32444-bib-0022] Maray, A. R. M., Mostafa, M. K., & Fakhrany, A. E. (2017). Effect of pretreatments and drying methods on physico‐chemical, sensory characteristics and nutritional value of oyster mushroom. Journal of Food Processing and Preservation, 42, e13352. 10.1111/jfpp.13352

[fsn32444-bib-0023] Mizgier, P., Kucharska, A. Z., Sokol, L. A., Kolniak, O. J., Kidon, M., & Fecka, I. (2016). Characterization of phenolic compounds and antioxidant and anti‐inflammatory properties of red cabbage and purple carrot extracts. Journal of Functional Foods, 21, 133–146. 10.1016/j.jff.2015.12.004

[fsn32444-bib-0024] Ning, X., Feng, Y., Gong, Y., Chen, Y., Qin, J., & Wang, D. (2019). Drying features of microwave and far‐infrared combination drying on white ginseng slices. Food Science and Biotechnology, 28, 1065–1072. 10.1007/s10068-018-00541-0 31275706 PMC6595014

[fsn32444-bib-0025] Omari, A., Behroozi, K. N., & Sharifian, F. (2018). Drying kinetic and artificial neural network modeling of mushroom drying process in microwave‐hot air dryer. Journal of Food Process Engineering, 41, e12849. 10.1111/jfpe.12849

[fsn32444-bib-0026] Pakonkiad, P., Nattapol, P., & Lamul, W. (2018). Control of microwave assisted macadamia drying. Journal of Microwave Power and Electromagnetic Energy, 52(1), 60–72. 10.1080/08327823.2017.1421872

[fsn32444-bib-0027] Podsedek, A. (2007). Natural antioxidants and antioxidant capacity of *Brassica* vegetables: A review. LWT ‐ Food Science and Technology, 40, 1–11. 10.1016/j.lwt.2005.07.023

[fsn32444-bib-0028] Rajkumar, G., Shanmugam, S., Galvao, M. D. S., Leite, M. T. S., Dutra, R. D., Mujumdar, A. S., & Narain, N. (2016). Comparative evaluation of physical properties and aroma profile of carrot slices subjected to hot air and freeze drying. Drying Technology, 35, 699–708. 10.1080/07373937.2016.1206925

[fsn32444-bib-0029] Razgeh, L. D., Fathabad, A. E., Shariatifar, N., Esmail, M., & Khaneghah, A. M. (2020). Effects of hot air and microwave drying on the phenolic components and physicochemical properties of unripe grapes (Qoura). Current Nutrition and Food Science, 16, 718–725. 10.2174/1573401315666190801105936

[fsn32444-bib-0030] Szewczyk, K., Bogucka, A., Vorobets, N., Grzywa, A., & Granica, S. (2020). Phenolic composition of the leaves of *Pyrola rotundifolia* L. and their antioxidant and cytotoxic activity. Molecules, 25(7), 1–16. 10.3390/molecules25071749 PMC7180938 32290223

[fsn32444-bib-0031] Wang, H., Liu, D., Yu, H., Wang, D., & Li, J. (2019). Optimization of microwave coupled hot air drying for Chinese yam using response surface methodology. Processes, 7, 745. 10.3390/pr7100745

[fsn32444-bib-0032] Wang, H., Zhang, M., & Mujumdar, A. S. (2014). Comparison of three new drying methods for drying characteristics and quality of shiitake mushroom (*Lentinus edodes*). Drying Technology, 32, 1791–1802. 10.1080/07373937.2014.947426

[fsn32444-bib-0033] Wang, Q., Li, S., Han, X., Ni, Y., Zhao, D., & Hao, J. (2019). Quality evaluation and drying kinetics of shitake mushrooms dried by hot air, infrared and intermittent microwave‐assisted drying methods. LWT ‐ Food Science and Technology, 107, 236–242. 10.1016/j.lwt.2019.03.020

[fsn32444-bib-0034] Wang, R. Y., Zhao, Y., Zhu, L. L., Fang, Z. X., & Shi, Q. L. (2020). Effect of carrier types on the physicochemical and antioxidant properties of spray‐dried black mulberry juice powders. Journal of Food Measurement and Characterization, 14, 1201–1212. 10.1007/s11694-019-00369-0

[fsn32444-bib-0035] Wang, Y., Li, X., Chen, X., Li, B., Mao, X., Miao, J., Zhao, C., Huang, L., & Gao, W. (2018). Effects of hot air and microwave‐assisted drying on drying kinetics, physicochemical properties, and energy consumption of chrysanthemum. Chemical Engineering and Processing ‐ Process Intensification, 129, 84–94. 10.1016/j.cep.2018.03.020

[fsn32444-bib-0036] Wiczkowski, W., Szawara, N. D., & Topolska, J. (2013). Red cabbage anthocyanins: Profile, isolation, identification, and antioxidant activity. Food Research International, 51(1), 303–309. 10.1016/j.foodres.2012.12.015

[fsn32444-bib-0037] Xia, Z. (2013). Anti‐browning of mushroom (*Agaricus bisporus*) slices by glutathione during hot air drying. Advance Journal of Food Science and Technology, 5, 1100–1104. 10.19026/ajfst.5.3213

[fsn32444-bib-0038] Xu, W., Song, C., Li, Z., Song, F., Hu, S., Li, J., Zhu, J., & Raghavan, G. S. (2018). Temperature gradient control during microwave combined with hot air drying. Biosystems Engineering, 169, 175–187. 10.1016/j.biosystemseng.2018.02.013

[fsn32444-bib-0039] Yadav, A., Nagda, N., & Singh, U. (2017). Effect of pretreatments, drying methods on mushroom‐ A review. Progressive Agriculture, 17, 261–265. 10.5958/0976-4615.2017.00046.1

[fsn32444-bib-0040] Yu, Q., Duan, J., Yu, N., & Fan, L. (2020). Enhancing the antityrosinase activity of saponins and polyphenols from asparagus by hot air coupled with microwave treatments. LWT ‐ Food Science and Technology, 124(4), e109174. 10.1016/j.lwt.2020.109174

[fsn32444-bib-0041] Zhang, F., Zhang, M., & Mujumdar, A. S. (2011). Drying characteristics and quality of restructured wild cabbage chips processed using different drying methods. Drying Technology, 29(6), 682–688. 10.1080/07373937.2010.525729

[fsn32444-bib-0042] Zhou, Y., Zhou, C., Pan, D., Wang, Y., & Cao, J. (2020). The effect of sodium chloride levels on the taste and texture of dry‐cured ham. Journal of Food Measurement and Characterization, 14(2), 2646–2655. 10.1007/s11694-020-00511-3

